# Macrophage activation syndrome as an unusual presentation of paucisymptomatic severe acute respiratory syndrome coronavirus 2 infection: A case report

**DOI:** 10.1097/MD.0000000000021570

**Published:** 2020-08-07

**Authors:** Sanaz Lolachi, Sarah Morin, Matteo Coen, Kaveh Samii, Alexandra Calmy, Jacques Serratrice

**Affiliations:** a Division of General Internal Medicine, Department of Medicine; b Hematology Division, Department of Oncology, Geneva University Hospitals; c University of Geneva, Faculty of Medicine, Unit of Development and Research in Medical Education (UDREM); d Laboratory of Virology and Division of Infectious Diseases, Geneva University Hospitals, Geneva, Switzerland.

**Keywords:** coronavirus, hydroxychloroquine, hyper-inflammation, interleukin-6, macrophages

## Abstract

**Rationale:**

Macrophage activation syndrome (MAS) is a rare life-threatening condition characterized by cytokine-mediated tissue injury and multiorgan dysfunction.

**Patient Concerns:**

We describe the unique case of young man who developed MAS as the sole manifestation of an otherwise paucisymptomatic severe acute respiratory syndrome coronavirus 2 (SARS-CoV-2) infection.

**Diagnoses:**

Clinical and biological criteria led to the diagnosis of MAS; cytokine profile was highly suggestive reverse transcription polymerase chain reaction for SARS-CoV-2 in nasopharyngeal swabs was negative, but serum anti-SARS-CoV-2 immunoglobulin A and immunoglobulin G resulted positive leading to the diagnosis of SARS-CoV-2 infection.

**Interventions:**

The patient was treated with empiric antibiotic and hydroxychloroquine.

**Outcomes:**

Clinical improvement ensued. At follow-up, the patient is well.

**Lesson:**

SARS-CoV-2 infection may trigger develop life-threatening complications, like MAS. This can be independent from coronavirus disease 2019 gravity.

## 1 Introduction

Macrophage activation syndrome (MAS) is a rare life-threatening condition characterized by cytokine-mediated tissue injury and multiorgan dysfunction.^[1]^ It can complicate Coronavirus Disease 2019 (COVID-19) pneumonia^.^^[2]^

Aims and scope of the report: to describe the unique case of a patient who developed MAS as the sole manifestation of a severe acute respiratory syndrome coronavirus 2 (SARS-CoV-2) infection, and to highlight that even paucisymptomatic COVID-19 patients can develop life-threatening complications.

## 2 Case report

A 16-year-old boy was admitted for investigation of fatigue evolving since 72 hours. He had no medical history, nor travel history and took no medications or drugs. Vitals signs as well as physical examination were normal, except for a temperature of 40°C, mild neck stiffness and palpable nuchal lymph nodes. Laboratory findings showed lymphopenia (0.61 G/L) and thrombocytopenia (70 G/L) without anaemia; C-reactive protein (CRP) was 49 mg/L (Upper Reference Limit, URL <5). Coagulation testing showed elevation of both the prothrombin time (49.6 second), and the partial thromboplastin time (34 sec) as well as mild hyperfibrinogenemia (fibrinogen 4.1 g/L; normal range: 1.5–3.5). Kidney and liver function tests were within normal ranges. Meningitis was suspected, but cerebrospinal fluid analysis was negative. Viral panel for hepatitis B virus, hepatitis C virus, cytomegalovirus, Epstein-Barr virus, Parvovirus B19, human herpes virus-6, and human immunodeficiency virus was negative, as well as toxoplasmosis serology. Reverse transcription polymerase chain reaction for severe acute respiratory syndrome coronavirus 2 (SARS-CoV-2) in 2 nasopharyngeal swabs was negative, but serum anti-SARS-CoV-2 immunoglobulin A and immunoglobulin G enzyme-linked immunosorbent assay (Euroimmun, Seekamp, Germany) resulted positive (immunoglobulin A and immunoglobulin G titers: 8.4 and 10.48, respectively).

Two days after admission, his condition worsened with sustained high fever, rigors, and hypotension. Repeated blood tests showed leukopenia (4.2 G/L), with worsening lymphopenia (0.29 G/L) and thrombocytopenia (40 G/L), as well as new-onset anemia (Hb 123 g/L). Liver enzymes were slightly elevated (SGOT [AST] 65 U/l, SGPT (ALT) 64 U/L respectively; URL <40) as well as lactate dehydrogenase (287 U/L; normal range 87–210), ferritin (1504 μg/L, normal range 11–340), and triglycerides (2.8 mmol/L; URL <2); high-sensitivity cardiac troponin-T (hs-cTn) levels were elevated (115 ng/L, URL 14 ng/L), without EKG modification. A part from increased D-Dimer (5360 ng/mL; URL <500), coagulation tests were not modified. Kidney function was normal. Both CRP and PCT were elevated (125.50 mg/L and 11.50 μg/L, respectively). Whole-body CT-scan was devoid of anomalies apart from hepatosplenomegaly. A treatment with ceftriaxone (2 gr once daily for 5 days) was empirically started; he was also given a single 800 mg dose of hydroxychloroquine (HCQ), according to the hospital's protocol. To note blood and urine cultures finally resulted sterile.

Rapid clinical deterioration with high, sustained fever, cytopenias, rising transaminases and ferritin, and evolving coagulopathy prompted us to suspect a MAS (or secondary hemophagocytic lymphohistiocytosis, HLH) in the context of SARS-CoV-2 infection. Bone marrow aspiration, biopsy and immunophenotyping by flow cytometry were performed. Monoclonal blast cell proliferation was absent, but some abnormalities in 3 monocytes and granulocytes maturation were noted; there was no evidence of hemophagocytosis. Serum immunoglobulin dosage and complement levels were within normal range, antinuclear antibodies were not detected. Serum cytokine dosage showed elevated levels of TNF-α (24.02 pg/mL; URL <4), IL-6 (226.81 pg/mL; URL <1.5), and IL-1 Ra (>10000 pg/mL; range 20–880), with normal IL-8 levels (57.51 pg/mL; URL <90). To note, reverse transcription polymerase chain reaction for SARS-CoV-2 in bone marrow cells resulted negative. With an HScore of 188 (70%–80% probability of a hemophagocytic syndrome; to note the optimal cut-off is 169), a diagnosis of MAS was suspected. Patient's condition and blood parameters rapidly improved and could be discharged on day 10. At 2 months follow-up, the patient is fine, without symptoms.

## 3 Discussion

We present an atypical case of a SARS-CoV-2 infection in a young man, characterized by clinical and biological features of MAS without respiratory symptoms. MAS is a rare life-threatening condition characterized by the uncontrolled hyper-activation of cytotoxic lymphocytes and macrophages, resulting in cytokine-mediated tissue injury and multiorgan dysfunction. Clinical features include high-grade persistent fever, hepatosplenomegaly, and lymphadenopathy. Laboratory features comprehend cytopenias, elevated CRP, coagulation abnormalities (hypofibrinogenemia, elevated D-dimer, prolonged prothrombin and partial thromboplastin time), evidence of hepatocellular injury, high levels of triglycerides and ferrritin, and hemophagocytosis (ie, phagocytosis by tissue-resident macrophages [histiocytes] of erythrocytes, leukocytes, platelets, and their precursors) on biopsy of bone marrow and other tissues.^[1]^ Signs of organ failure can appear in the advanced stages of the syndrome. In the adult, it typically develops on the background of inflammatory diseases such as neoplasms (frequently T-cell lymphomas), autoimmune diseases or infections (mainly viral, notable the Epstein-Barr virus).^[2]^ The gold standard for the diagnosis of MAS is the HScore, a diagnostic tool based on 9 criteria (clinical: underlying immunosuppression, high temperature, hepatosplenomegaly; biological: cytopenias, hypertriglyceridemia, hyperferrritinemia, elevated SGOT [AST], hypofibrinogenemia; and cytological: hemophagocytosis features on bone marrow aspirates).^[3]^ Despite its name (HLH), hemophagocytosis is not pathognomonic, since it is presence is neither necessary nor sufficient for MAS diagnosis.^[1]^

It has been recently highlighted that hyper-inflammation in COVID-19 pneumonia can represent a novel MAS-like pathology; moreover, there are several similarities between COVID-19 and MAS, notably in terms of cytokine profile.^[4]^ It is worth noticing that ours is a most peculiar case of a paucisymptomatic COVID-19 patient who developed MAS as the sole manifestation of SARS-CoV-2 infection.^[5]^ Apart from the diagnostic criteria, cytokine profile in our patient was characterized by high levels of circulating cytokines (TNF-α and IL-6) and natural cytokine-inhibitors (IL-1 Ra), both features being highly suggestive of this hyper-inflammatory state.^[6]^ When MAS is associated to infections, like in our case, glucocorticoids can be offered in addition to specific anti-microbiological treatment.^[5]^ Glucocorticoid treatment in COVID-19 is still controversial.^[4]^ Thus, besides empiric antibiotic therapy, our patients received HCQ. Before the results of the multinational registry analysis were published,^[7]^ this anti-malarial drug was considered a potential candidate for treatment of COVID-19.^[8]^ Moreover, HCQ has an effect on cytokine production by decreasing the secretion of IL-6 and TNF-α which has been associated to a lowered MAS risk in patients with systemic lupus erythematosus.^[9]^

## 4 Conclusions

Our case highlights that SARS-CoV-2 infection may trigger MAS even in the absence of COVID-19 pneumonia, o rather that paucisymptomatic COVID-19 can lead to life-threatening complications. Indeed, COVID-19 is much more than a severe acute respiratory syndrome!

Parental informed written consent was indeed being obtained for the purpose of publication of the case details.

## Author contributions

Sanaz Lolachi: Conception of the work, Data collection, drafting of the article.

Sarah Morin: Conception of the work, Data collection.

Matteo Coen: data analysis and interpretation, drafting of the article, critical revision of the article.

Kaveh Samii: data analysis and interpretation, critical revision of the article.

Alexandra Calmy: data analysis and interpretation, critical revision of the article

Jacques Serratrice: data analysis and interpretation, critical revision of the article.
